# Supplementary material for: Safe engineering of CAR T cells for adoptive cell therapy of cancer using long‐term episomal gene transfer
Source: EMBO Mol Med. 2016 May 6;8(7):702–11. doi: 10.15252/emmm.201505869 (PMC4931286; doi:10.15252/emmm.201505869)
Supplement: Supplementary file 1 — Appendix [file EMMM-8-702-s001.pdf]

Safe engineering of CAR T cells for adoptive cell therapy of cancer using long-term episomal gene transfer

Chuan Jin, Grammatiki Fotaki, Mohanraj Ramachandran, Berith Nilsson, Magnus Essand, Di Yu

Appendix

Table of Contents

Page 2..... Appendix Figure S1
Page 3..... Appendix Figure S2
Page 4..... Appendix Figure S3
Page 5..... Appendix Figure S4
Page 6..... Appendix Figure S5
Page 7..... Appendix Figure S6
Page 8..... Appendix Table S1

Appendix Figure S1

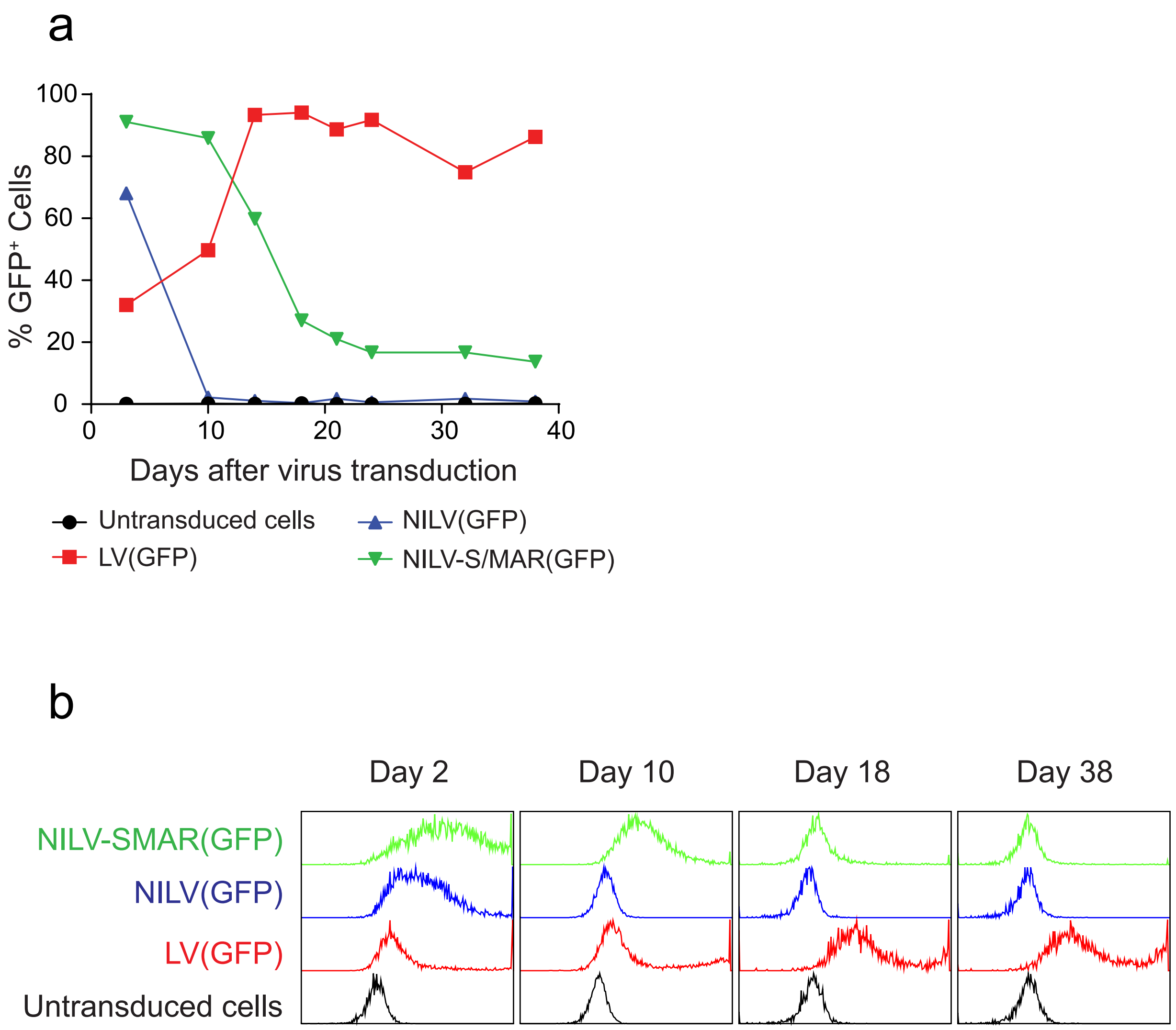

Appendix Figure S1. Validation of NILV-S/MAR vector for long-term transgene expression. 911 cells were transduced with GFP-expressing conventional LV vector, integrase-deficient NILV vector or NILV vector with an S/MAR element and cultured under normal condition for up to 38 days, without puromycin selection. GFP expression was monitored over time by flow cytometry. Both long-term transgene expression (a) and the expression level (b) are shown.

Appendix Figure S2

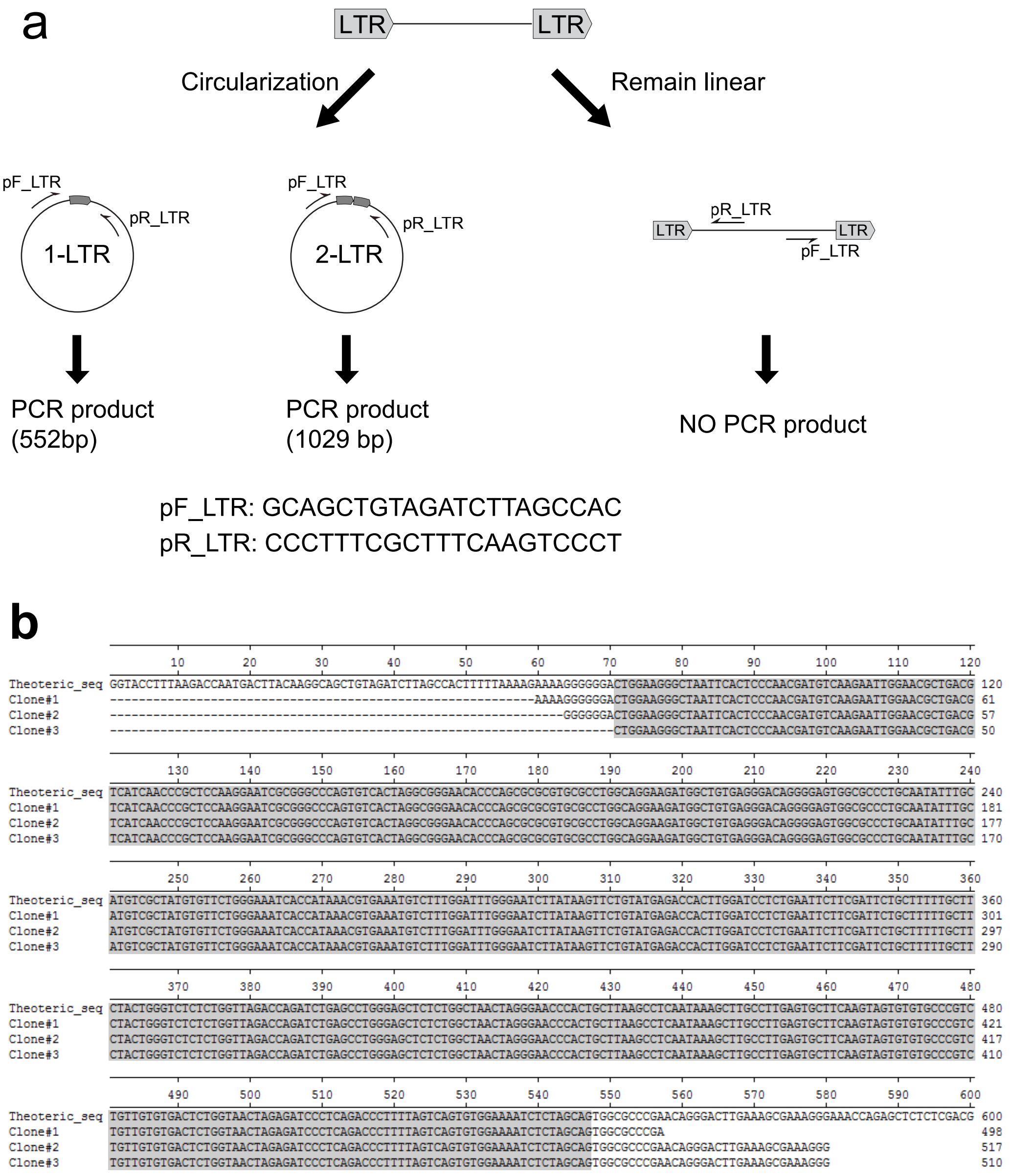

Appendix Figure S2. PCR assay to demonstrate circularization and the formation of LTR junction from lentiviral vectors. a) Illustration of the experimental design to verify circularization and the formation of LTR junction. A specific primer pair (pF_LTR and pR_LTR) was designed to PCR-amplify the head-tail circularized joint region of DNA circles. A junction formation containing one LTR (1-LTR) yields a PCR product of 552 bp while a junction formation containing two LTRs (2-LTR) yields a PCR product of 1029 bp. No PCR product will be formed for un-joined LTRs. b) Amplified PCR products from 3 different clones were sequenced and aligned with the theoretical 1-LTR junction region (gray highlighted) showing perfect match. This proved the formation of only the 1-LTR circle, as is also observed by the 552 bp band in Figure 1d.

Appendix Figure S3

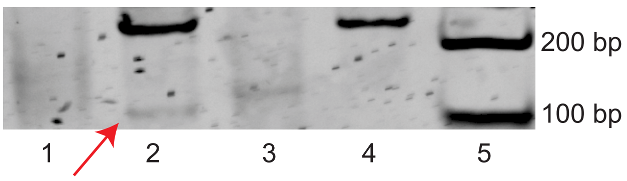

1. Untransduced cells
2. LV(GFP)
3. NILV(GFP)
4. NILV-S/MAR(GFP)
5. 1kb+ DNA Marker

Appendix Figure S3. Verification of genome insertion events using Lam-PCR. A549 cells were transduced with the different viral vectors and culture for approximately 4 weeks. DNA from these cells were collected and subjected to LAM-PCR essentially follow the published step-by-step protocol (1). The amplified DNA was separated by 1% agarose gel and imaged. The band at size 225 bp corresponds to the internal control (that the proviral DNA sequence is present in the sample). The narrow bands at approximately 100 bp (red arrow) correspond to insertional events. Weak bands in lanes 1 and 3 at about 125 bp are suspected to be unspecific products because of absence of the control band at 225 bp. We conclude that the insertional events of NILV-S/MAR vector are below detection level or not the typical lentiviral LTR-LTR precision insertion.

Reference:
1. Schmidt M, Schwarzwaelder K, Bartholomae C, Zaoui K, Ball C, Pilz I, Braun S, Glimm H, von Kalle C (2007) High-resolution insertion-site analysis by linear amplification-mediated PCR

Appendix Figure S4

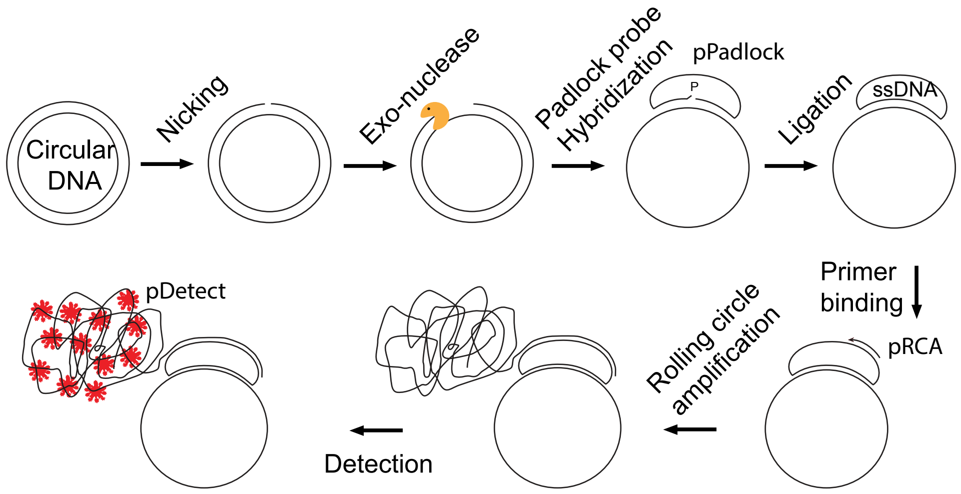

pPadlock: $^{PO_4-}$GCAACCCCAAATCCCC**CAGTGAATGCGAGTCCGTCTAAG**AGAGTAGTACAGCAGCCGTCAAAT GAGTTTTCCAGA
pRCA: GACGGCTGCTGTACTACTCT
pDetect: CTTAGACGGACTCGCATTCACTG^{-Cy3}

Appendix Figure S4. The principal steps involved in padlock hybridization and rolling circle amplification for the detection of DNA circles. Cells were transduced with the different lentiviral vectors and maintained for more than 10 passages (>30 days) before seeded on glass slides. The cells were then permeabilized and treated with DNA nickase to nick one of the two strands of circular DNA followed by exo-nuclease to digest the nicked DNA strand. A padlock probe (pPadlock) was applied for hybridization to the single-stranded template DNA in the presence of DNA ligase. Only hybridized padlock probes form a short single-stranded DNA (ssDNA) circle on the template DNA. The ssDNA circle is further amplified through rolling circle amplification (RCA) by phi29 polymerase and the pRCA primer. The RCA product is then detected by a Cy3-labeled detection probe (pDetect), which is complementary to part of the padlock probe. Bold letters in pPadlock indicate the binding site of the Cy3-labeled detection probe (pDetect) and underlined letters indicate the binding site for rolling circle amplification primer (pRCA).

Appendix Figure S5

a b

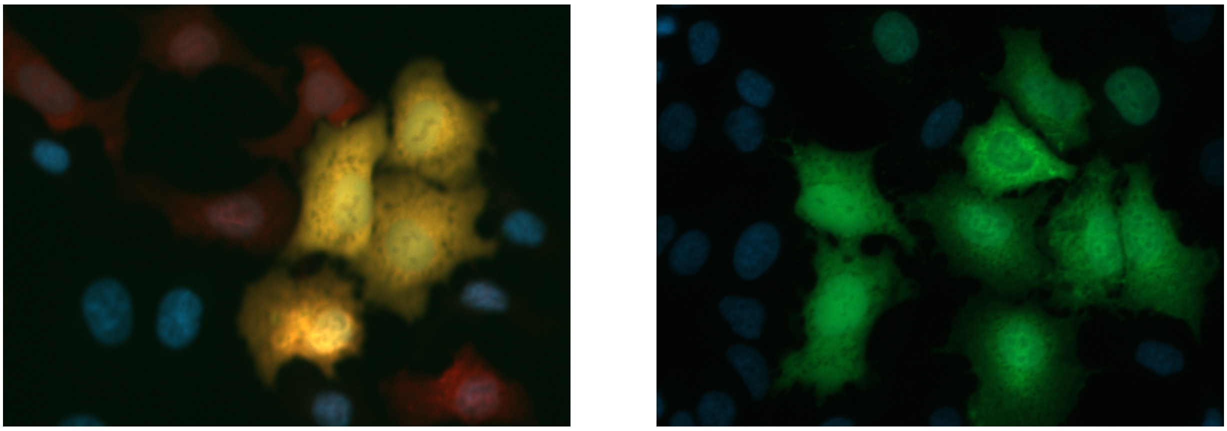

A549/RFP$^+$Luc$^+$
NILV-S/MAR(GFP, shNT)

A549/RFP$^+$Luc$^+$
NILV-S/MAR(GFP, shRFP)

Appendix Figure S5. NILV-S/MAR-mediated gene knockdown of RFP. A549 cells were engineered to stable express RFP and Luciferase (Figure 2b). The cells were then transduced with either NILV-S/MAR(GFP, shNT) or NILV-S/MAR(GFP, shRFP), selected on puromycin and then kept in culture for up to 44 days. Representative microscopy images showing cell nuclei stained with Hoechst 33342 and the expression of GFP in cells transduced with (a) NILV-S/MAR(GFP, shNT) and (b) NILV-S/MAR(GFP, shRFP). Cells in (a) also express RFP and therefore appears yellow while RFP expression has been knocked down in (b). Images were taken using Zeiss AxioImager M2 (Zeiss, Germay).

Appendix Figure S6

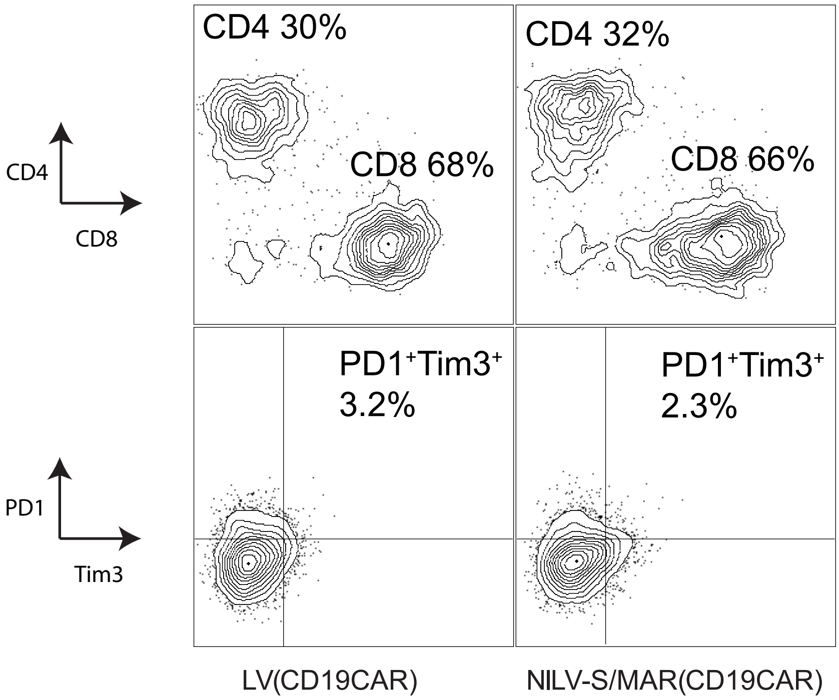

Appendix Figure S6. Phenotype of CD19 CAR T cells after expansion. T cells were transduced with LV(CD19CAR) or NILV-S/MAR(CD19CAR), expanded for 12 days and analyzed by flow cytometry as indicated in Figure 3B. CD4/CD8 cell ratios and expression of PD1 and Tim3 are presented as density plots from one representative donor. Double positivity of PD1 and Tim3 is seen as a sign of T cell exhaustion. These experiments were performed for 6 donors without detecting any differences in phenotype between LV(CD19CAR) and NILV-S/MAR(CD19CAR)-engineered T cells.

Appendix Table S1

Statistic p-values

Figure 2d Day 20

Tukey's multiple comparisons test	Mean Diff.	95% CI of diff.	Significant?	Summary	Adjusted P Value
A549 vs. A549/RFP+Luc+	-1.45E+06	-1.612e+006 to -1.279e+006	Yes	****	< 0.0001
A549 vs. A549/RFP+Luc+/shNT	-1.72E+06	-1.888e+006 to -1.555e+006	Yes	****	< 0.0001
A549 vs. A549/RFP+Luc+/shRFP	-308833	-475290 to -142376	Yes	**	0.0016
A549/RFP+Luc+ vs. A549/RFP+Luc+/shNT	-275729	-442186 to -109272	Yes	**	0.0032
A549/RFP+Luc+ vs. A549/RFP+Luc+/shRFP	1.14E+06	970141 to 1.303e+006	Yes	****	< 0.0001
A549/RFP+Luc+/shNT vs. A549/RFP+Luc+/shRFP	1.41E+06	1.246e+006 to 1.579e+006	Yes	****	< 0.0001

Figure 2d Day 44

Tukey's multiple comparisons test	Mean Diff.	95% CI of diff.	Significant?	Summary	Adjusted P Value
A549 vs. A549/RFP+Luc+	-1.65E+06	-1.767e+006 to -1.529e+006	Yes	****	< 0.0001
A549 vs. A549/RFP+Luc+/shNT	-1.64E+06	-1.759e+006 to -1.522e+006	Yes	****	< 0.0001
A549 vs. A549/RFP+Luc+/shRFP	-393184	-511947 to -274421	Yes	****	< 0.0001
A549/RFP+Luc+ vs. A549/RFP+Luc+/shNT	7823	-110940 to 126586	No	ns	0.9964
A549/RFP+Luc+ vs. A549/RFP+Luc+/shRFP	1.26E+06	1.136e+006 to 1.374e+006	Yes	****	< 0.0001
A549/RFP+Luc+/shNT vs. A549/RFP+Luc+/shRFP	1.25E+06	1.128e+006 to 1.366e+006	Yes	****	< 0.0001

Figure 3e CD107 expression

Tukey's multiple comparisons test	Mean Diff.	95% CI of diff.	Significant?	Summary	Adjusted P Value
NILV-S/MAR(MOCK) vs. LV(CD19CAR)	-12.73	-15.81 to -9.660	Yes	****	< 0.0001
NILV-S/MAR(MOCK) vs. NILV(CD19CAR)	-1.65	-4.723 to 1.423	No	ns	0.4544
NILV-S/MAR(MOCK) vs. NILV-S/MAR(CD19CAR)	-11.53	-14.61 to -8.460	Yes	****	< 0.0001
LV(CD19CAR) vs. NILV(CD19CAR)	11.08	8.010 to 14.16	Yes	****	< 0.0001
LV(CD19CAR) vs. NILV-S/MAR(CD19CAR)	1.2	-1.873 to 4.273	No	ns	0.6978
NILV(CD19CAR) vs. NILV-S/MAR(CD19CAR)	-9.883	-12.96 to -6.810	Yes	****	< 0.0001

Figure 3f IFN gamma expression

Tukey's multiple comparisons test	Mean Diff.	95% CI of diff.	Significant?	Summary	Adjusted P Value
NILV-S/MAR(MOCK) vs. LV(CD19CAR)	-414.5	-763.3 to -65.64	Yes	*	0.0163
NILV-S/MAR(MOCK) vs. NILV(CD19CAR)	85.93	-262.9 to 434.7	No	ns	0.8999
NILV-S/MAR(MOCK) vs. NILV-S/MAR(CD19CAR)	-272.5	-621.3 to 76.36	No	ns	0.1612
LV(CD19CAR) vs. NILV(CD19CAR)	500.4	151.6 to 849.2	Yes	**	0.0035
LV(CD19CAR) vs. NILV-S/MAR(CD19CAR)	142	-206.8 to 490.8	No	ns	0.6702
NILV(CD19CAR) vs. NILV-S/MAR(CD19CAR)	-358.4	-707.2 to -9.572	Yes	*	0.0427

Figure 3l Average tumor growth

Tukey's multiple comparisons test	Mean Diff.	95% CI of diff.	Significant?	Summary	Adjusted P Value
Mock T cells vs. LV(CD19CAR)	169.7	8.168 to 331.2	Yes	*	0.038
Mock T cells vs. NILV-SMAR(CD19CAR)	200	26.04 to 373.9	Yes	*	0.0216
LV(CD19CAR) vs. NILV-SMAR(CD19CAR)	30.27	-140.4 to 200.9	No	ns	0.9

Figure 3m Survival Curves

Log-rank (Mantel-Cox) test	Chi square	df	P value
All groups	9.442	2	0.0089
Mock vs LV	6.521	1	0.0107
Mock vs NILV-S/MAR	6.378	1	0.0116
LV vs NILV-S/MAR	0.002527	1	0.9599
Bonferroni-corrected threshold			0.0167

Figure 3q Quantification of T cell infiltration

Tukey's multiple comparisons test	Mean Diff.	95% CI of diff.	Significant?	Summary	Adjusted P Value
Mock T cells vs. LV(CD19CAR)	-781.5	-1106 to -456.6	Yes	****	< 0.0001
Mock T cells vs. NILV-SMAR(CD19CAR)	-684.2	-1009 to -359.3	Yes	***	0.0003
LV(CD19CAR) vs. NILV-SMAR(CD19CAR)	97.31	-227.6 to 422.2	No	ns	0.7106
