# Supplementary material for: Safe engineering of CAR T cells for adoptive cell therapy of cancer using long‐term episomal gene transfer
Source: EMBO Mol Med. 2016 May 6;8(7):702–11. doi: 10.15252/emmm.201505869 (PMC4931286; doi:10.15252/emmm.201505869)
Supplement: Supplementary file 3 — Source Data for Figure 1 [file EMMM-8-702-s002.pdf]

Source Data for Fig 1d

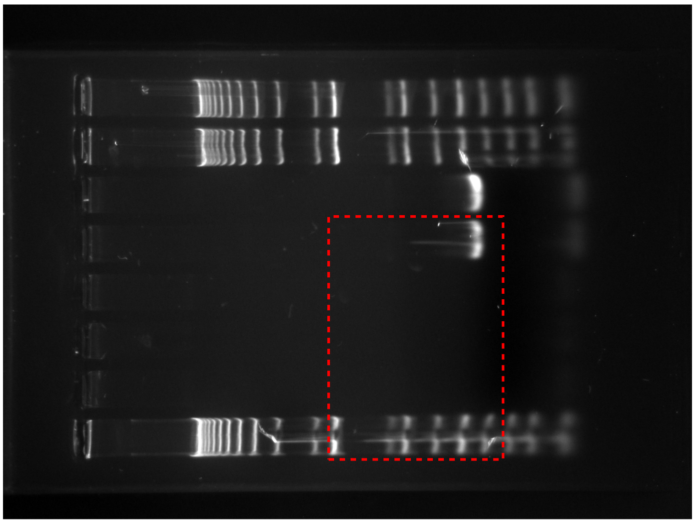

Cropped and used in Figure 1 d

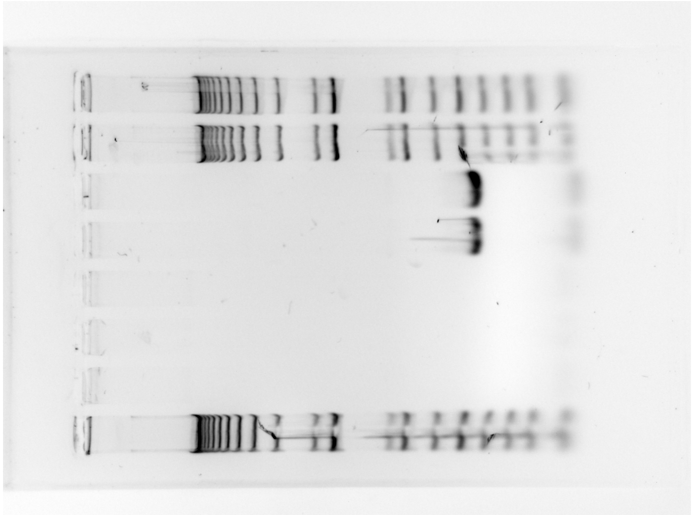

Alternative view (Inverted color)

Source Data for Fig 1e

Southen Blot #1

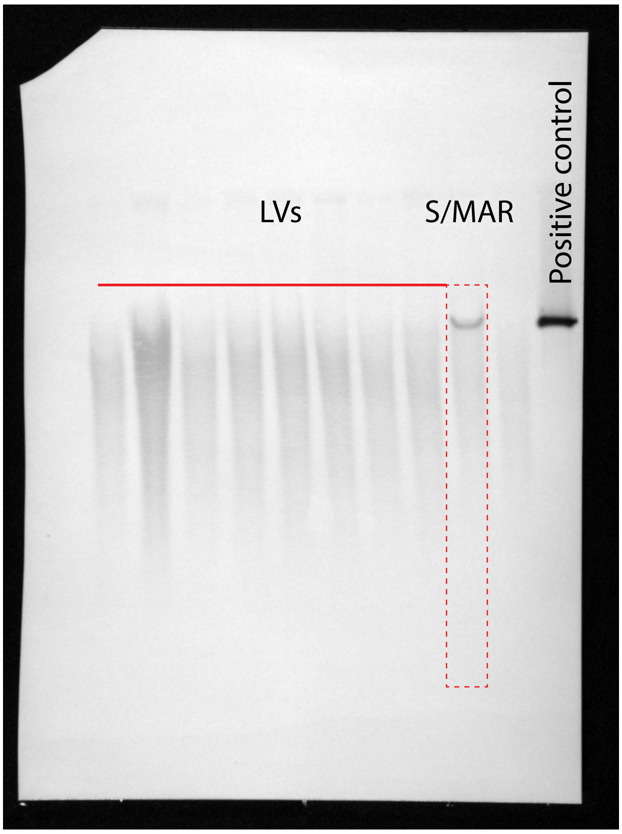

Southen Blot #2

1kb+ marker

Positive control

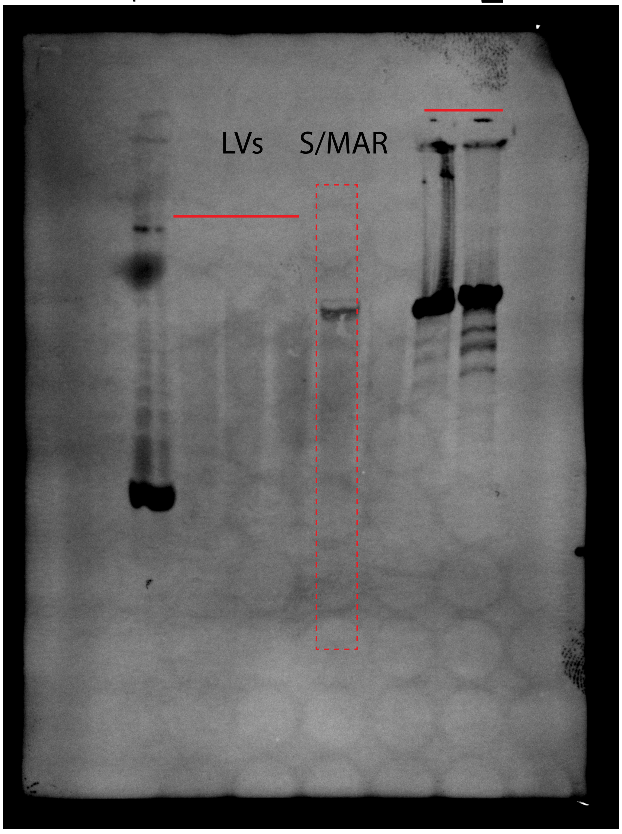

The 1650 band in1kb+ size marker is visulized by probing the plasmid ori region (pUC)
